# Anthropometric factors, physical activity, and the risk of soft tissue sarcoma: a case–control study

**DOI:** 10.1097/CEJ.0000000000001016

**Published:** 2026-03-23

**Authors:** Federica Turati, Giovanna Esposito, Margherita Pizzato, Paola Bertuccio, Carlotta Galeone, Claudia Santucci, Ilaria Milanesi, Giovanni Corso, Piero Lovreglio, Prisco Piscitelli, Fabio Parazzini, Francesca Bravi, Eva Negri, Sofia Pavanello, Carlo La Vecchia

**Affiliations:** a Department of Clinical Sciences and Community Health, Department of Excellence 2023–2027, University of Milan; b Fondazione IRCCS Ca’ Granda Ospedale Maggiore Policlinico, Milan; c Department of Medical and Surgical Sciences, University of Bologna, Bologna; d Department of Public Health, Experimental and Forensic Medicine, Unit of Hygiene, University of Pavia, Pavia; e Bicocca Applied Statistics Center (B-ASC), University of Milano-Bicocca; f Division of Breast Surgery, European Institute of Oncology IRCCS; g Department of Oncology and Hemato-Oncology, University of Milan, Milan; h Section of Occupational Medicine, Interdisciplinary Department of Medicine, University of Bari Aldo Moro, Bari; i Laboratory of Interdisciplinary Research Applied to Medicine (DReAM), Local Health Authority ASL Le, Lecce; j Department of Psychology and Health Sciences, Pegaso University, Naples; k Department of Cardiac, Thoracic, and Vascular Sciences and Public Health, University of Padua; l Occupational Medicine Unit, University Hospital of Padua; m Centre of Studies and Activities for Space CISAS ‘G. Colombo’ of University of Padua, Padua, Italy

**Keywords:** anthropometric factors, body mass index, case–control, epidemiology, physical activity, risk factors soft tissue sarcoma, waist circumference, waist-to-hip ratio

## Abstract

**Background:**

Soft tissue sarcomas (STSs) are rare malignancies with largely unknown etiology; the role of anthropometry and physical activity has been rarely explored.

**Methods:**

We analyzed 2011–2019 data of an Italian, multicenter, hospital-based case–control study including 498 incident, histologically confirmed STS cases and 969 controls. Self-reported height, weight, and physical activity data were collected; hip and waist circumferences were measured in 76% of cases and 61% of controls. Odds ratios (ORs) and 95% confidence intervals (CIs) were estimated using multiple-adjusted logistic regression models.

**Results:**

Compared with BMI < 25 kg/m^2^, the ORs for BMI one year prior to diagnosis/interview were 1.05 (95% CI: 0.80–1.37) for 25–29.9 kg/m^2^ and 1.28 (95% CI: 0.90–1.83) for ≥30 kg/m^2^. The OR was increased for BMI 25–29.9 kg/m^2^ at age 30; no association emerged for BMI at age 50 years. For waist circumference, the ORs were 1.93 (95% CI: 1.38–2.70) for >102 cm in males or >88 cm in females, and 3.54 (95% CI: 2.40–5.22) for the highest versus the lowest tertile. For waist-to-hip ratio, the ORs were 1.18 (95% CI: 0.81–1.71) for the intermediate, and 2.81 (95% CI: 1.95–4.03) for the highest tertile. Subjects reporting ≥5 h/week of total or intermediate/high intensity leisure-time physical activity at age 30–39 years had ORs of 0.63 (95% CI: 0.40–1.00) and 0.56 (95% CI: 0.32–1.00), respectively.

**Conclusion:**

These findings show a positive association between abdominal adiposity and STS risk, and an inverse association with regular leisure-time physical activity.

## Introduction

Soft tissue sarcomas (STSs) are a heterogeneous group of malignant mesenchymal tumors, including a large number of histological subtypes (Sbaraglia *et al*., 2021). STSs are rare in adults, accounting for less than 1% of all malignant tumors (Mastrangelo *et al*., 2012), but are proportionally more frequent in children, adolescents and young adults (Aben *et al*., 2012; Steliarova-Foucher *et al*., 2017). Despite advances in molecular and clinical characterization, the etiology of most STS cases remains largely unknown. Established risk factors are few and include exposure to ionising radiation (IARC, 2012a), certain chemicals, such as selected phenoxy herbicides and chlorophenols (IARC, 2012b; Edwards *et al*., 2021), and rare inherited cancer predisposition syndromes (Connolly *et al*., 2025). However, these factors are likely to account for only a minor proportion of cases, and the role of other environmental factors, including lifestyle-related factors, remains largely unclear (Burningham *et al*., 2012).

Anthropometric characteristics and physical activity have been associated with the risk of several common cancers through pathways involving hormonal regulation, inflammation, and immune function (Lauby-Secretan *et al*., 2016). Yet, their potential influence on STS development has been rarely explored (Tavani *et al*., 1999; Moore *et al*., 2016; Sun *et al*., 2024).

To contribute to an enhanced understanding of potentially preventable determinants of STS, we investigated the associations of selected anthropometric factors and measures of physical activity with STS risk in a large case–control study conducted in Italy.

## Methods

### Study population

A hospital-based case–control study on STS has been ongoing since 2011 in Northern Italy. The current analysis comprises 498 incident STS cases and 969 controls recruited between 2011 and 2019 in six hospitals located in the Metropolitan City of Milan and nearby areas.

Cases were identified at the National Cancer Institute (*n* = 493, 99%) and the European Institute of Oncology (*n* = 5, 1%) in Milan. Controls were selected from patients admitted to major general hospitals of the greater Milan area.

Eligible cases were adults aged 18–85 years (median age = 57) with a newly diagnosed (i.e. diagnosed within 2 years; median time between diagnosis and interview <1 month), histologically confirmed STS (International Classification of Diseases for Oncology, 3^rd^ edition, ICD-O-3, codes listed in Table 1), who had been residing in Italy for at least 15 years prior to enrollment. Patients with Kaposi’s sarcoma, any previous cancer, or severe neurologic or psychiatric disorders were excluded.

**Table 1:** Distribution of cancer subtypes in 498 soft tissue sarcoma cases and of selected socio-demographic and lifestyle characteristics in 498 cases and 969 controls^a^. Italy, 2011–2019

	Cases No. (%)	Controls No. (%)
Age		
<40	76 (15.3)	139 (14.3)
40–44	55 (11.0)	66 (6.8)
45–49	40 (8.0)	66 (6.8)
50–54	54 (10.8)	66 (6.8)
55–59	52 (10.4)	90 (9.3)
60–64	69 (13.9)	120 (12.4)
65–69	59 (11.9)	137 (14.1)
70–74	47 (9.4)	154 (15.9)
≥75	46 (9.2)	131 (13.5)
Sex		
Male	288 (57.8)	529 (54.6)
Female	210 (42.2)	440 (45.4)
Years of education		
<7	88 (17.7)	183 (19.0)
7–11	158 (31.8)	333 (34.6)
12–14	146 (29.4)	327 (34.0)
≥15	105 (21.1)	119 (12.4)
Smoking		
Never	219 (44.0)	413 (42.7)
Ex	163 (32.7)	268 (27.7)
Current (cigarettes/day)		
<10	35 (7.0)	75 (7.8)
10–19	51 (10.2)	131 (13.5)
≥20	30 (6.0)	80 (8.3)
Alcohol drinking (drinks/week)		
Never	106 (21.3)	173 (18.0)
<7	146 (29.3)	282 (29.3)
7–13	88 (17.7)	173 (18.0)
14–20	86 (17.3)	208 (21.6)
≥21	72 (14.5)	126 (13.1)
Sarcoma subtype (ICD-O-3 histology codes)		
Liposarcoma (8850–8858)	168 (33.7)	
Fibrosarcoma (8810–8815)	69 (13.9)	
Leiomyosarcoma (8890)	43 (8.6)	
Dermatofibrosarcoma (8832)	41 (8.2)	
GIST (8936)	27 (5.4)	
Synovial (9040–9043)	20 (4.0)	
MPNST (9540–9560)	15 (3.0)	
Chondrosarcoma (9220–9240)	12 (2.4)	
Angiosarcoma (9120, 9124, 9130, 9133, 9170)	10 (2.0)	
Sarcoma NOS (8800–8805)	57 (11.4)	
Other types^b^	36 (7.2)	
Sarcoma location		
Limbs	248 (49.8)	
Trunk	241 (48.4)	
Head and neck	9 (1.8)	

GIST, gastrointestinal stromal tumor; ICD-O-3, International Classification of Diseases for Oncology, 3^rd^ edition; MPNST, malignant peripheral nerve sheath tumor; NOS, not otherwise specified.

a The sum may do not add up to the total because of missing values.

b Including rhabdomyosarcoma (*n* = 9, 1.8%, ICD-O-3 codes: 8900, 8901, 8912), Ewing sarcoma (*n* = 7, 1.4%, ICD-O-3 code: 9260), perivascular epithelioid cell neoplasm (*n* = 5, 1%, ICD-O-3 code: 8714), myofibroblastic sarcoma (*n* = 4, 0.8%, ICD-O-3 code: 8825), chordoma (*n *= 4, 0.8%, ICD-O-3 code: 9370), malignant fibrous histiocytoma (*n* = 2, 0.4%, ICD-O-3 code: 8830), alveolar soft part sarcoma (*n* = 2, 0.4%, ICD-O-3 code: 9581), malignant granular cell soft tissue tumor (*n* = 1, 0.2%, ICD-O-3 code: 9580), carcinosarcoma (*n* = 1, 0.2%, ICD-O-3 code: 8982), and clear cell sarcoma NOS (*n* = 1, 0.2%, ICD-O-3 code: 9044).

Controls were adults aged 18–85 years (median age = 62) admitted for a variety of acute, nonneoplastic conditions. Specifically, 21.8% were hospitalized for trauma, 16.5% for nontraumatic orthopedic problems, 53.3% for acute surgical diseases, and 8.4% for other acute conditions, such as disorders of the eye, ear, or skin. Patients whose primary reason for hospital admission was a chronic condition or a disease potentially associated with tobacco use, alcohol consumption, or long-term changes in diet were not eligible. Refusal rates were below 10% for both cases and controls.

The study was conducted in accordance with the Declaration of Helsinki and was approved by the Ethics Committees of the National Cancer Institute, Milan (INT 32/09 of 11 June 2009), and of the Gaetano Pini Hospital (n. 5752 of 09 June 2010). All participants provided written informed consent.

### Data collection

Cases and controls were interviewed in hospital by the same centrally trained interviewers using a standardized questionnaire. The questionnaire collected information on socio-demographic characteristics, anthropometric measures, lifestyle habits including physical activity, lifetime tobacco smoking and alcohol drinking, dietary habits in the 2 years before diagnosis/interview, a problem oriented medical history, family history of cancer, and, for females, gynecological and obstetric data, and history of use of oral contraceptives and hormone replacement therapy.

With regard to anthropometric data, subjects were asked to report their height and weight one year before cancer diagnosis or interview, as well as at ages 30 and 50 years. BMI was computed as weight divided by squared height (kg/m^2^). In 966 subjects (376 cases, 76%; and 590 controls, 61%), the interviewers measured waist circumference (2 cm above the umbilicus) and hip (maximal protrusion) circumference. The waist-to-hip ratio (WHR) was calculated as waist circumference divided by hip circumference. Categories of waist circumference and WHR were defined according to sex-specific tertiles. Additionally, waist circumference was dichotomized based on the WHO risk-based cutoffs (>102 cm in males and >88 cm in females) (WHO, 2011). As only a few participants had WHR values below the WHO thresholds (0.90 for males and 0.85 for females), we also created a dichotomous variable using a cutoff of 1.0, which indicates that waist circumference exceeds hip circumference.

The section on physical activity included questions on self-reported occupational and leisure-time physical activity at different ages (12, 15–19, 30–39, and 50–59 years). Subjects classified their occupational physical activity level as very heavy, heavy, intermediate, standing, or sedentary, according to a structured scale classified into 5 categories (scores from 1 to 5 for increasing activity levels). In the analysis, the scores of the two highest levels were combined due to the small number of subjects in the highest activity levels. Leisure-time physical activity was assessed as the total number of hours per week spent in activities such as sports, cycling, and gardening, categorized according to low, intermediate, and high intensity. The total weekly hours of activity, as well as the hours spent in activities of intermediate or high intensity, were calculated. Information on the specific types of sports practiced was also collected.

### Statistical analysis

We estimated the odds ratios (ORs) for STS, with the corresponding 95% confidence intervals (CIs), according to anthropometric and physical activity measures using unconditional logistic regression models including terms for age (in categories, <40, 40–44, 45–49, 50–54, 55–59, 60–64, 65–69, 70–74, ≥75), sex, area of residence, and years of education (in categories, <7, 7–11, 12–14, ≥15). The few missing values for education (one among cases and seven among controls) were imputed to the median value according to case–control status. In additional sensitivity analyses, we further adjusted the ORs for tobacco smoking and alcohol drinking. Tests for trend were performed by modeling the variables as ordinal. Statistical analyses were performed with SAS version 9.4 (SAS Institute, Cary, NC, USA) and R software (version 4.5.1, 13 June 2025, R Foundation for Statistical Computing, Vienna, Austria) for Windows.

## Results

Table 1 shows the distribution of STS subtypes in cases, and of selected socio-demographic and lifestyle characteristics in cases and controls. Compared with controls, STS cases were slightly younger and more educated, while the distribution of sex, smoking habits, and alcohol consumption was relatively similar across groups. Liposarcoma was the most common STS histologic type (33.7%), followed by fibrosarcoma (13.9%), leiomyosarcoma (8.6%), and dermatofibrosarcoma (8.2%); 11.4% of cases were unclassified STSs. Nearly half of STSs arose in the limbs (49.8%), 48.4% in the trunk, and 1.8% in the head and neck.

Table 2 provides the ORs of STS according to selected anthropometric measures. No clear pattern of association emerged between BMI and STS risk. Compared with subjects with BMI < 25 kg/m^2^, the OR was 1.05 (95% CI: 0.80–1.37) for those with BMI of 25–29.9 kg/m^2^ and 1.28 (95% CI: 0.90–1.83) for those with BMI ≥ 30 kg/m^2^ (*P*-trend = 0.208). A modest excess risk was observed for BMI of 25–29.9 kg/m^2^ at age 30 years (OR = 1.48, 95% CI: 1.10–1.99), but not for BMI ≥ 30, while no association emerged with high BMI at age 50 years. Higher abdominal adiposity was associated with an increased risk. Males with waist circumference > 102 cm and females with waist circumference > 88 cm had an OR of 1.93 (95% CI: 1.38–2.70) compared with those below these cutoffs, and subjects in the highest sex-specific waist circumference tertile had an OR of 3.54 (95% CI: 2.40–5.22; *P*-trend <0.001). Similarly, a positive trend was observed for WHR (*P*-trend <0.001), with an OR of 2.81 (95% CI: 1.95–4.03) in the highest tertile compared with the lowest, and an OR of 1.38 (95% CI: 0.94–2.03) for a WHR > 1. Similar OR estimates were obtained after further adjustment for tobacco smoking and alcohol drinking. Specifically, the OR for BMI ≥ 30 kg/m^2^ before diagnosis was 1.19 (95% CI: 0.83–1.71); for waist circumference > 102 cm in males and >88 cm in females, 1.91 (95% CI: 1.35–2.69); for the highest sex-specific waist circumference tertile, 3.44 (95% CI: 2.32–5.12); and for the highest WHR tertile, 2.82 (95% CI: 1.95–4.07).

**Table 2:** Distribution of 498 soft tissue sarcoma cases and 969 controls^a^, and corresponding adjusted odds ratios (OR) with 95% confidence intervals (CI), according to various anthropometric measures

	Cases No. (%)	Controls No. (%)	OR^b^ (95% CI)
BMI one year prior to diagnosis/interview (kg/m^2^)			
<25	215 (43.3)	446 (46.0)	1.00
25–29.9	199 (40.0)	379 (39.1)	1.05 (0.80–1.37)
≥30	83 (16.7)	144 (14.9)	1.28 (0.90–1.83)
* P*-trend			*0.208*
BMI at age 50 years (kg/m^2^)^c^			
<25	93 (29.5)	219 (32.0)	1.00
25–29.9	149 (47.3)	314 (45.8)	1.11 (0.79–1.57)
≥30	73 (23.2)	152 (22.2)	0.98 (0.65–1.49)
* P*-trend			*0.988*
BMI at age 30 years (kg/m^2^)^d^			
<25	285 (62.2)	639 (70.6)	1.00
25–29.9	127 (27.7)	195 (21.5)	1.48 (1.10–1.99)
≥30	46 (10.0)	71 (7.8)	1.12 (0.72–1.76)
* P*-trend			*0.093*
WC (cm)			
M ≤ 102, F ≤ 88	262 (69.7)	471 (79.8)	1.00
M > 102, F > 88	114 (30.3)	119 (20.2)	1.93 (1.38–2.70)
WC (cm), sex-specific tertiles			
I	68 (18.1)	232 (39.3)	1.00
II	139 (37.0)	184 (31.2)	2.87 (1.94–4.25)
III	169 (44.9)	174 (29.5)	3.54 (2.40–5.22)
* P*-trend			*<0.001*
WHR			
≤1	54 (14.4)	124 (21.0)	1.00
>1	322 (85.6)	466 (79.0)	1.38 (0.94–2.03)
WHR, sex-specific tertiles			
I	90 (23.9)	230 (39.0)	1.00
II	110 (29.3)	212 (35.9)	1.18 (0.81–1.71)
III	176 (46.8)	148 (25.1)	2.81 (1.95–4.03)
* P*-trend			*<0.001*

BMI, body mass index; F, female; M, male; WC, waist circumference; WHR, waist-to-hip ratio.

a The sum may not add up to the total because of missing values.

b Adjusted for age, sex, area of residence, and education.

c Among subjects aged ≥50 years only.

d Among subjects aged ≥30 years only.

Table 3 shows the results for occupational and leisure-time physical activity. No clear association emerged for occupational activity at age 30–39 or 50–59 years, although a two-fold excess risk was noted among subjects in the third occupational activity category (intermediate level) at age 50–59 years (OR = 2.06, 95% CI: 1.11–3.82). Leisure-time physical activity tended to be inversely associated with STS risk. In particular, individuals engaging in ≥5 h/week of total and intermediate/high intensity activity at age 30–39 years had ORs respectively of 0.63 (95% CI: 0.40–1.00) and 0.56 (95% CI: 0.32–1.00). These ORs became 0.66 (95% CI: 0.37–1.18) and 0.62 (95% CI: 0.31–1.23), respectively, after further adjustment for waist circumference.

**Table 3:** Distribution of 498 soft tissue sarcoma cases and 969 controls^a^, and corresponding adjusted odds ratios (OR) with 95% confidence intervals (CI), according to occupational and leisure-time physical activity

	Cases No. (%)	Controls No. (%)	OR^b^ (95% CI)
Occupational physical activity			
At age 30–39 years			
1 (lowest)	143 (34.0)	254 (32.4)	1.00
2	180 (42.9)	364 (46.4)	0.96 (0.70–1.31)
3	42 (10.0)	54 (6.9)	1.58 (0.94–2.65)
4–5 (highest)	55 (13.1)	112 (14.3)	0.86 (0.54–1.38)
At age 50–59 years			
1 (lowest)	73 (28.1)	157 (29.2)	1.00
2	120 (46.2)	276 (51.4)	1.14 (0.76–1.72)
3	33 (12.7)	39 (7.3)	2.06 (1.11–3.82)
4–5 (highest)	34 (13.1)	65 (12.1)	1.04 (0.56–1.92)
Leisure-time physical activity, total number of hours/week			
At age 12 years			
0	361 (72.5)	756 (78.0)	1.00
1–4	103 (20.7)	137 (14.1)	1.34 (0.97–1.85)
≥5	34 (6.8)	76 (7.8)	0.76 (0.47–1.23)
At age 15–19 years			
0	312 (62.7)	596 (61.5)	1.00
1–4	133 (26.7)	253 (26.1)	0.84 (0.63–1.11)
≥5	53 (10.6)	120 (12.4)	0.81 (0.55–1.19)
At age 30–39 years			
0	308 (65.7)	571 (62.1)	1.00
1–4	128 (27.3)	252 (27.4)	1.02 (0.77–1.34)
≥5	33 (7.0)	96 (10.4)	0.63 (0.40–1.00)
At age 50–59 years			
0	222 (67.9)	443 (63.5)	1.00
1–4	79 (24.2)	182 (26.1)	1.19 (0.84–1.68)
≥5	26 (8.0)	73 (10.5)	0.78 (0.46–1.31)
Leisure-time physical activity, number of hours of intermediate and high intensity activity/week			
At age 12 years			
0	369 (74.1)	768 (79.3)	1.00
1–4	99 (19.9)	131 (13.5)	1.37 (0.98–1.90)
≥5	30 (6.0)	70 (7.2)	0.73 (0.44–1.21)
At age 15–19 years			
0	337 (67.7)	677 (69.9)	1.00
1–4	123 (24.7)	189 (19.5)	1.05 (0.78–1.42)
≥5	38 (7.6)	103 (10.6)	0.68 (0.44–1.06)
At age 30–39 years			
0	363 (77.4)	713 (77.6)	1.00
1–4	87 (18.6)	144 (15.7)	1.12 (0.80–1.55)
≥5	19 (4.1)	62 (6.7)	0.56 (0.32–1.00)
At age 50–59 years			
0	277 (84.7)	586 (84.0)	1.00
1–4	35 (10.7)	84 (12.0)	1.01 (0.64–1.61)
≥5	15 (4.6)	28 (4.0)	1.11 (0.55–2.24)

a The sum may do not add up to the total because of missing values.

b Adjusted for age, sex, area of residence, and education.

Figure 1 displays the results for the most frequently practiced sports. STS risk was higher among individuals who had ever practiced basketball/volleyball or tennis, while no associations were observed for other sports.

**Fig. 1:**
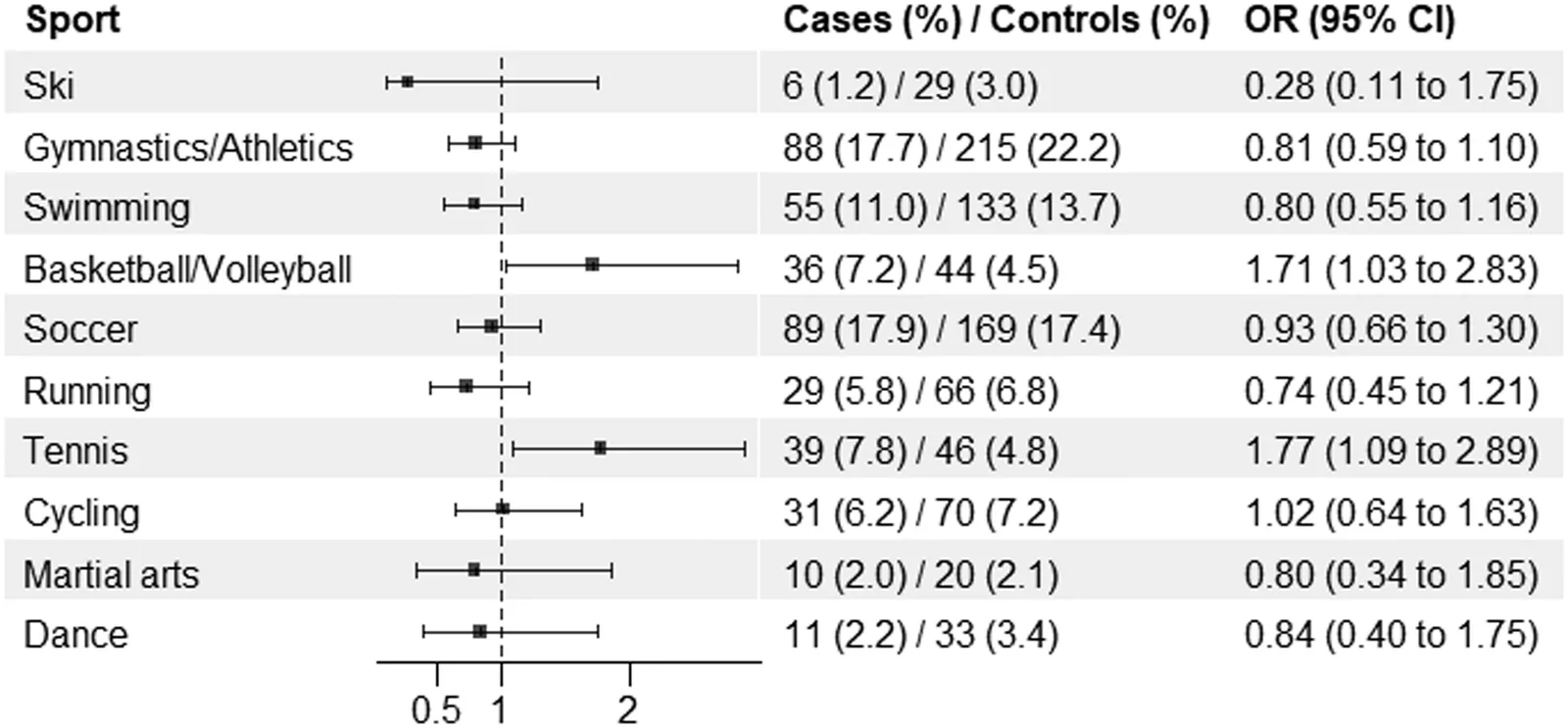
Adjusted odds ratios^a^ (OR) of soft tissue sarcoma, with 95% confidence intervals (CI), according to the most frequently practiced sports. ^a^Adjusted for age, sex, area of residence, and education.

Exploratory analyses by STS histologic type (liposarcoma, fibrosarcoma, leiomyosarcoma, and dermatofibrosarcoma) and broad categories of anatomical sites (upper or lower limbs, and trunk), showed no substantial differences in the associations with waist circumference, WHR, and leisure-time physical activity at 30–39 years (data not shown). Among the main sports, the association with tennis was similar for limb and trunk sarcomas, whereas that with basketball/volleyball appeared somewhat stronger for tumors of the trunk.

## Discussion

In this large study conducted in northern Italy, indicators of abdominal adiposity were positively associated with STS risk, whereas no clear pattern of association emerged for BMI, suggesting that fat distribution rather than overall body mass has a role in STS development. No association was observed with occupational physical activity, while a decreased risk was found in subjects engaging in ≥5 h/week of total or intermediate/high intensity leisure-time activity at age 30–39 years.

Our findings are in line with those from recent investigations indicating that abdominal adiposity may play a more relevant role in the risk of selected cancers than overall body adiposity as measured by BMI (De Ridder *et al*., 2016; Barberio *et al*., 2019; Sun *et al*., 2025). Increased waist circumference and WHR are indicators of abdominal fat accumulation and may better reflect metabolic complications of obesity. Abdominal fat mass is metabolically active and associated with chronic inflammation, insulin resistance, and hormonal dysregulation, which may promote carcinogenesis (Hardy *et al*., 2012; Renehan *et al*., 2015; Crudele *et al*., 2021).

Only a few studies have examined the association between anthropometric factors and STS risk, all (Zahm *et al*., 1989; Tavani *et al*., 1999; Sun *et al*., 2024) but one (Wagner *et al*., 2014) suggesting a positive relationship with BMI, including a large pooled analysis of Swedish cohorts based on over 2000 STS incident cases [the Obesity and Disease Development Sweden study] (Sun *et al*., 2024). Notably, an analysis based on a smaller dataset of the Obesity and Disease Development Sweden study (Sun *et al*., 2025), including 270 STSs, reported a stronger association with waist circumference than with BMI, with hazard ratios (HRs) of 1.23 (95% CI: 1.05–1.44) and 1.13 (95% CI: 0.98–1.28) per SD increase in waist circumference and BMI, respectively. To our knowledge, that is the only previous study which has examined indicators of abdominal obesity in relation to STS risk.

In the present study, we also examined BMI at ages 30 and 50 years to explore the potential role of adiposity earlier in adulthood and to reduce the likelihood of reverse causation, particularly for measures close to diagnosis that may have been influenced by preclinical disease. However, these age-specific measures relied on long-term recall and are therefore prone to additional measurement error. Moreover, the time interval between BMI at a given age and STS diagnosis (or index date among controls) varied across participants, resulting in heterogeneity in exposure timing that may complicate the biological interpretation of these findings.

We found an inverse association between leisure-time physical activity and STS risk, which only slightly attenuated after additional adjustment for abdominal adiposity. This suggests that physical activity may lower STS risk through mechanisms other than body weight reduction, potentially involving pathways related to inflammation, immune function, and oxidative stress, as for other cancers (Friedenreich *et al*., 2010). The association between physical activity and STS risk has been rarely explored. No association was found with leisure-time physical activity in a pooled analysis of 12 prospective U.S. and European cohorts including 851 STS cases (Moore *et al*., 2016). The HR for highest (90^th^ percentile) versus lowest (10^th^ percentile) levels of leisure-time physical activity was 0.94 (95% CI: 0.67–1.31), which minimally varied after adjustment for BMI (OR = 0.97, 95% CI: 0.70–1.34).

This is the first study examining specific sports in relation to STS. We observed excess risks among subjects who had ever practiced basketball/volleyball or tennis. As a possible, speculative, explanation, these activities involve repetitive localized mechanical stress and microtrauma to tendons, ligaments, or peri-articular soft tissues, potentially promoting chronic inflammation and tissue regeneration processes. However, we did not observe stronger associations for STS of the limbs compared with those of the trunk. Moreover, no clear duration–risk pattern emerged. For basketball/volleyball, the ORs were 1.66 (95% CI: 0.93–2.94) for ≤10 years and 1.79 (95% CI: 0.65–4.93) for >10 years of practice; for tennis, the corresponding ORs were 2.45 (95% CI: 1.01–5.95) and 1.91 (95% CI: 1.04–3.49). Results were, in any case, based on small numbers and warrant further investigation.

This study is among the largest epidemiological studies conducted so far on this rare malignancy. Its main strengths include the high participation rate and the availability of extensive data on anthropometric indicators, physical activity, and several other factors. To enhance comparability between cases and controls and reduce the likelihood of selection bias, we selected controls from major teaching and general hospitals in the study area, and excluded patients admitted for chronic conditions. All analyses were adjusted for area of residence to account for potential geographic variation in socio-demographic characteristics. Additional adjustment for tobacco smoking and alcohol consumption in sensitivity analyses did not materially modify the risk estimates.

Among the limitations, information on weight and height was self-reported. Subjects, particularly those overweight, tend to underestimate their weight and overestimate their height. A greater underreporting of weight among cases is possible and may have attenuated an association with BMI. However, given the similar interview setting for cases and controls, and the limited awareness of any hypothesis linking anthropometric factors to STS, a major differential misreporting appears unlikely. Similarly, data on physical activity were self-reported and collected using structured scales that have not been formally validated; therefore, some degree of misclassification cannot be excluded. However, the standardized interview procedure and the hospital setting likely enhanced comparability between cases and controls, reducing the likelihood of differential misclassification. Further, waist circumference and hip measurements were not available for 24% of cases and 39% of controls. However, in the overall sample, median BMI in subjects with a measured waist circumference was not substantially different from that in subjects for whom the information on waist circumference was missing (25.5 versus 26.0 kg/m^2^ among cases, and 25.5 and 25.0 kg/m^2^ among controls). Finally, we cannot exclude residual confounding by socio-demographic or other lifestyle factors. However, STS has no major known environmental correlates, and the results for waist circumference and WHR remained similar after further adjustment for BMI, as did those for leisure-time physical activity after accounting for abdominal adiposity.

In conclusion, this investigation adds to the limited knowledge on risk factors for STS. Our results support a positive association with abdominal adiposity and suggest an inverse association with leisure-time physical activity.

## Acknowledgements

The study was funded by the European Union – Next Generation EU – National Recovery and Resilience Plan (NRRP) MC62 – Investment 2.1 Enhancement and strengthening of biomedical research in the NHS (PNRR-TR1-2023-12377497), and also supported by European Union – Next Generation EU, in the context of the National Recovery and Resilience Plan, Investment PE8 – Project Age-It: ‘Ageing Well in an Ageing Society’ (DM 1557 11 October 2022). FT was supported by the Research Support Plan 2022–2023 from the University of Milan.

The authors thank Dr. Fabio Levi for his assistance with the histological classification of soft tissue sarcomas.

C.L.V., C.G., F.T. and E.N. conceptualized and designed the study; C.L.V., C.G., E.N., F.T., M.P., G.E. and F.B. developed the study methodology; F.T., M.P., F.B., G.E., P.B., C.S. and I.M. performed data cleaning and validation, and prepared the final analysis dataset; M.P. reviewed histological records and assigned International Classification of Diseases for Oncology (ICD-O, 3^rd^ edition) codes; F.T. analyzed the data and drafted the manuscript; S.P., F.B., F.P., P.L., and P.P. acquired funding for the project; all authors read and approved the final manuscript.

### Conflicts of interest

There are no conflicts of interest.
